# Frequency, Timing, and Prediction of Major Bleeding Complications From Percutaneous Renal Biopsy

**DOI:** 10.1177/2054358120923527

**Published:** 2020-05-25

**Authors:** Melissa Schorr, Pavel S. Roshanov, Matthew A. Weir, Andrew A. House

**Affiliations:** 1 Division of Nephrology, Department of Medicine, Schulich School of Medicine & Dentistry, Western Ontario, London, Canada; 2 London Health Sciences Centre, Victoria Hospital, London, Ontario, Canada; 3 Department of Epidemiology and Biostatistics, Western University, London, Ontario, Canada

**Keywords:** percutaneous renal biopsy, complications, timing, bleeding, risk prediction, native kidney, transplant kidney

## Abstract

**Background and Objectives:**

The risk and timing of bleeding events following ultrasound-guided percutaneous renal biopsy are not clearly defined.

**Design setting, participants, and measurements:**

We performed a retrospective study of 617 consecutive adult patients who underwent kidney biopsy between 2012 and 2017 at a tertiary academic hospital in London, Canada. We assessed frequency and timing of minor (not requiring intervention) and major (requiring blood transfusion, surgery, or embolization) bleeds and developed a personalized risk calculator for these.

**Results:**

Bleeding occurred in 79 patients (12.8%; 95% confidence interval [CI]: 10.4%-15.7%). Minor bleeding occurred in 67 patients (10.9%; 95% CI: 8.6%-13.6%). Major bleeding occurred in 12 patients (1.9%; 95% CI: 1.1%-3.4%); 2 required embolization or surgery (0.3%; 95% CI: 0.09%-1.2%) and 10 required blood transfusion (1.6%; 95% CI: 0.9%-3.0%). Seventy-three of 79 events were identified immediately on post-procedure ultrasound (92.4% of cases; 95% CI: 84.4%-96.5%). Four of 617 patients experienced a minor event not detected immediately (0.6%; 95% CI: 0.3%-1.7%). Two patients (0.3%; 95% CI: 0.09%-1.2%) suffered a major complication that was not recognized immediately; both required blood transfusions only. There were no deaths or nephrectomies. A risk calculator using age, body mass index, platelet count, hemoglobin concentration, size of the target kidney, and whether the kidney is native, or an allograft predicted minor (C-statistic, 0.70) and major bleeding (C-statistic, 0.83).

**Conclusions:**

This retrospective study of 617 patients who had percutaneous ultrasound-guided renal biopsies supports the safety of short post-biopsy monitoring for most patients. A risk calculator can further personalize estimates of complication risk (http://perioperativerisk.com/kbrc).

## Abrégé

**Contexte:**

Les risques d’hémorragie et le moment où surviennent ces événements après une biopsie rénale percutanée échoguidée ne sont pas clairement définis.

**Méthodologie:**

Nous avons procédé à une étude rétrospective sur 617 patients adultes consécutifs ayant subi une biopsie rénale entre 2012 et 2017 dans un center hospitalier universitaire de soins tertiaires de London, au Canada. Nous avons analysé la fréquence des saignements et le moment où est survenue une hémorragie mineure (ne nécessitant aucune intervention) ou majeure (nécessitant une transfusion sanguine, une chirurgie ou une embolisation). Un calculateur de risque a ultérieurement été développé pour chacun.

**Résultats:**

Des 617 patients étudiés, 79 (12,8 %; IC 95 % : 10,4 %-15,7 %) ont vécu un événement hémorragique mineur (n=67 [10,9 %]; IC 95 % : 8,6 %-13,6 %) ou majeur (n=12 [1,9 %]; IC 95 % : 1,1 %-3,4 %). Deux hémorragies majeures ont nécessité une embolisation ou une chirurgie (0,3 %; IC 95 % : 0,09 %-1,2 %), les dix autres ayant été traitées par transfusion sanguine (1,6 %; IC 95 % : 0,9 %-3,0 %). Des 79 événements rapportés, 73 ont été détectés à l’échographie immédiatement après l’intervention (92,4 % des cas; IC 95 % : 84,4 %-96,5 %). Quatre patients (0,6 %; IC 95 % : 0,3 %-1,7 %) ont subi une complication mineure n’ayant pas été détectée immédiatement après la biopsie. Deux patients (0,3 %; IC 95 % : 0,09 %-1,2 %) ont souffert d’une complication majeure n’ayant pas été reconnue immédiatement, mais qui n’a nécessité que des transfusions sanguines. Aucun décès ou néphrectomie n’a été rapporté. Un calculateur du risque d’hémorragie utilisant l’âge, l’indice de masse corporelle, la numération plaquettaire, le taux d’hémoglobine, la taille du rein biopsié, et tenant compte du fait que le rein est natif ou une allogreffe, a pu prédire un événement mineur (statistique C = 0,70) ou majeur (statistique C = 0,83).

**Conclusion:**

Cette étude rétrospective sur 617 patients confirme qu’il serait prudent d’assurer une surveillance de courte durée pour la plupart des patients qui subissent une biopsie percutanée échoguidée. L’emploi d’un calculateur du risque d’hémorragie précise davantage le risque de complications estimé (http://perioperativerisk.com/kbrc).

## What was known before

Renal biopsy is a diagnostic and prognostic procedure that has been used for more than 50 years in the evaluation of kidney disease. The reported frequency and timing of complications associated with renal biopsy is variable and the appropriate duration of post-procedure monitoring remains controversial.

## What this adds

Our study retrospectively evaluated percutaneous, ultrasound-guided, adult renal biopsies. Our results demonstrate that major bleeding complications are uncommon and often identified immediately post-procedure. Patient-specific risk of minor and major bleeding could be estimated before biopsy with a risk calculator developed from these data. Our study supports shorter post-biopsy monitoring times in the outpatient setting and allows for patient-specific bleeding risks to be determined for both native and allograft biopsies.

## Introduction

Renal biopsy is an important procedure in determining renal disease etiology and prognosis, guiding its management and monitoring renal transplant function and viability.^1-5^ Automated biopsy instruments and real-time ultrasound guidance have improved safety^6^ since the procedure was first described in 1951,^7^ but there remains a risk of bleeding, which in some cases can be significant.

Many biopsies are now performed on outpatients with discharge home on the day of the procedure.^8-10^ The reported frequency and timing of complications associated with renal biopsy is variable and the appropriate duration of post-procedure monitoring remains controversial,^8,10-18^ with some suggesting that patients must be monitored for 24 hours to avoid missing serious complications.^19-24^

We set out to (1) assess the type, frequency, and timing of kidney biopsy–related bleeding events at our center; (2) assess risk factors for complications and develop a calculator for estimating individual patient risk of bleeding; and (3) determine the optimal post-biopsy monitoring time for outpatient procedures.

## Methods

### Reporting and Ethics

We have reported this study according to the Transparent Reporting of a multivariable prediction model for Prognosis or Diagnosis (TRIPOD) statement.^25^ As a quality improvement project, this study met criteria for a waiver of review by the Research Ethics Board at Western University, London, Ontario, Canada.

### Study Design, Source of Data, and Participants

We conducted a retrospective chart review of all adult patients who underwent biopsy of a native kidney or allograft between 2012 and 2017 at London Health Science Center’s University Hospital, a 415-bed tertiary-care center in Southwestern Ontario. Patients who underwent more than one biopsy during this 5-year period were included as separate events each time. We felt this was justified as many of the risk factors are variable over time (such as platelet count and hemoglobin) and the indications for and circumstances during the biopsy could also be potentially quite different. For example, someone may have undergone a native renal biopsy during a presentation with nephrotic syndrome and subsequently had a transplant allograft biopsy a number of years later.

We abstracted data on demographics and risk factors for bleeding (Table 1), which we defined *a priori*. We collected clinical and laboratory characteristics without blinding to patients’ complication status.

**Table 1.** Patient Characteristics Before and After Imputation of Missing Data.

	N (% of total) or median [IQR]
**Age, years, median [IQR]**	57.0 [46.0, 66.0]
**Female**	225 (36.5%)
**Male**	392 (63.5%)
**Platelet count before biopsy, median [IQR]**	203.0 [157.0, 256.0]^a^
**Hemoglobin before biopsy, g/L, median [IQR]**	108.0 [91.0, 125.0]^b^
**Size of biopsied kidney, cm, median [IQR]**	11.5 [10.7, 12.5]^c^
**Body mass index, kg/m^2^, median [IQR]**	27.9 [24.4, 32.0]^d^
**Serum urea, mmol/L, median [IQR]**	13.2 [8.9, 19.7]^e^
**Serum creatinine, μmol/L, median [IQR]**	201.0 [135.0, 347.0]^f^
**Native (vs allograft) kidney biopsy**	247 (40.0%)^g^
**INR >1.3 before biopsy**	16 (2.6%)^h^
**Proteinuria (dipstick 3+ or UACR >300 or >3.5 g/day)**	126 (20.4%)^i^
**Inpatient biopsy**	260 (42.1%)
**History of diabetes**	462 (74.9%)
**History of hypertension**	165 (26.7%)
**Etiology of kidney disease**	
**Diabetes mellitus**	77 (12.5%)
**Hypertension, vascular, or ischemic**	46 (7.5%)
**Glomerulonephritis or vasculitis**	154 (25.0%)
**Congenital or inherited**	87 (14.1%)
**None, other, or unknown**	253 (41.0%)
**Indication**	
**Acute kidney injury (non-transplant)**	39 (6.3%)
**Hematuria and/or non-nephrotic proteinuria**	116 (18.8%)
**Nephrotic proteinuria**	51 (8.3%)
**Graft dysfunction (excluding delayed graft function)**	324 (52.5%)
**Delayed graft function**	21 (3.4%)
**Chronic kidney disease of unknown etiology**	11 (1.8%)
**Paraprotein**	40 (6.5%)
**Kidney living donor**	10 (1.6%)
**Other**	5 (0.8%)

*Note.* Footnotes: Data are post imputation. Missing data were imputed where specified. BMI = body mass index; INR = international normalized ratio; UACR = urine albumin-to-creatinine ratio.

a Missing data imputed for 2 patients (0.3%).

b Missing data imputed for 2 patients (0.3%).

c Missing data imputed for 59 patients (9.6%).

d Missing data imputed for 64 patients (10.4%).

e Missing data imputed for 22 patients (3.6%).

f Missing data imputed for 1 patient (0.2%).

g Missing data imputed for 3 patients (0.5%).

h Missing data imputed for 19 patients (3.2%).

i missing data imputed for 213 patients (34.5%); for missing data regarding proteinuria, we assumed that the value would have been less than 3+ on dipstick, <300 mg/mmol for urine albumin-to-creatinine ratio, and <3.5 g/day for a 24-hour urine collection.

### Peri-Procedure Management

The standard practice at our center for pre-biopsy includes holding anticoagulation and antiplatelet medications—warfarin is held for 5 days and antiplatelets for 7 days. Baseline bloodwork includes hemoglobin, platelets, international normalized ratio (INR), creatinine, and urea. Platelets of <100, hemoglobin <70, and INR >1.5 are all treated and rechecked prior to the procedure; if these cut-offs are not met, the procedure is delayed or rescheduled. Neither desmopressin acetate (DDAVP) nor conjugated estrogen is routinely used. Blood pressure is measured the day of the procedure and if it is greater than 160 systolic or 90 diastolic, the procedure is postponed until better control is achieved. Our standard of practice for all renal biopsies is to scan the kidney immediately post procedure. A large or expanding perinephric hematoma would be an indication for close follow-up with repeat ultrasound within 2 hours of the procedure. Progression to computed tomography (CT) angiography would be in consultation with our Radiology colleagues. Prior to this study, outpatients were kept in the radiology post-procedure care room for the remainder of the day—usually 5 hours—with a repeat hemoglobin looking for significant (ie, >10 g/L) drop from prior measure. Prior to discharge, they are reassessed by a Nephrology staff or trainee and a decision regarding need for admission is made.

Inpatients are monitored on the ward, kept supine for 5 hours post procedure, and have a repeat complete blood count (CBC) checked after the 4 hours. A hemoglobin decrease of >10 g/L, symptomatic hypotension, or persistent pain would prompt an urgent repeat ultrasound. Gross hematuria in isolation does not instigate further investigation.

### Biopsy Procedures

All biopsies were performed for clinical indications and according to usual practice at our center. In total, during our study period, there were 6 different Nephrologists who performed biopsies. Individuals performing biopsies were either staff nephrologists or fellows in training under direct supervision of an experienced attending physicians. In all cases, the BARD Monopty 18-gauge, 16 cm Disposable Core Biopsy Instrument was used (C. R. Bard Inc, Tempe AZ, USA), which is a spring-loaded biopsy instrument that provides a 22 mm core of tissue. All procedures were carried out under real-time ultrasound guidance with a needle guide mounted to the ultrasound transducer. Operators identified adequate biopsy sites with input from ultrasound technicians and obtained at least 2 cores of tissue deemed adequate by an on-scene pathology technician; no more than 5 attempts were made to obtain these samples. For native kidney biopsies, only the lower pole was targeted. For renal allografts, the lower pole was the commonest target, but mid-pole and upper pole targets were used when dictated by individual anatomy. Operators and ultrasound technicians scanned the kidney after each pass to detect bleeding. Immediate post-biopsy hematomas are identified by the nephrologist and ultrasound technician as a perinephric echogenic fluid collection. Doppler ultrasound can show rapid, active bleeding. Subsequent scans are performed and interpreted by our Radiology colleagues. For patients with a post-biopsy hematoma, pressure was applied for 10 minutes followed by immediate repeat scanning. Those with ongoing bleeding underwent repeat ultrasound or computed tomographic angiogram after consultation with Interventional Radiology. Patients without any evidence of bleeding remained supine for 4 hours post-procedure and were monitored for 5 hours, after which outpatients were discharged home.

### Outcomes

We reviewed medical records to identify complications occurring up to 1 week after biopsy. We defined a minor event as a perinephric hematoma, gross hematuria, or bleeding that did not require transfusion or intervention. That is, the presence of gross hematuria, or a perinephric hematoma, or a drop in hemoglobin of <10 g/L (with no evidence of ongoing bleeding on repeat imaging) that did not result in hemodynamic instability or necessitate medical intervention such as blood transfusion, embolization, or nephrectomy. We defined major complications as bleeding requiring transfusion, surgical intervention, or embolization of the bleeding vessel.

### Patient Characteristics

Guided by prior evidence,^1,11,20,26-29^ we collected data on characteristics that may influence the risk of bleeding including age, platelet count, hemoglobin concentration, kidney size, body mass index (BMI), severity of kidney dysfunction, coagulation status, the type of kidney (native or allograft), comorbidities, number of passes with the biopsy needle, and the indications for biopsy.

### Statistical Analysis

We performed all analyses using R version 3.5.1. The Supplementary Appendix provides additional detail of the statistical analysis.

#### Approach to missing data

We imputed missing data 100 times using multiple imputations with predictive mean matching for predictor variables.

#### Development of the kidney biopsy risk calculator

We performed this analysis according to guidance for the development of prediction models in small datasets.^30^ We selected 6 predictor variables based on the following criteria: (1) clinical plausibility or evidence of association with kidney biopsy-related bleeding in prior research, (2) sufficiency of data (such that, for categorical variables, each cell in a contingency table with the primary outcome would have at least 10 patients), and (3) reliable measurement in routine clinical care without significant confounding by dialysis (eg, serum creatinine and urea are reliably measured, but are not reflective of renal function in patients receiving dialysis). The following 6 predictors met our inclusion criteria: age, platelet count, hemoglobin concentration, BMI, biopsy of a native kidney (versus an allograft), and size of the biopsied kidney in its greatest dimension on ultrasound examination. We fit a logistic regression model on the composite outcome of any biopsy-related bleeding events using penalized maximum likelihood estimation with 7.8 degrees of freedom (for 10.3 outcome events per degree of freedom).^31^ We kept continuous variables continuous and modeled their association with the outcome using restricted cubic spline functions. We included all variables in the model simultaneously regardless of statistical significance. We performed internal validation in 1000 bootstrap samples and calculated average bias-corrected C-statistics (a measure of a model’s ability to discriminate between patients who develop an outcome and those who do not), calibration slope, calibration intercept, and calibration curves (measures of how closely predicted risks match the observed probability of an outcome event). We then used the same approach to fit penalized logistic regression models that predict (1) only minor events based on the predicted log-odds of any complication from the composite outcome model and (2) only major complications (requiring transfusion or intervention) based on the pre-biopsy hemoglobin (assuming a linear form) and the predicted log-odds of any complication from the composite outcome model.

## Results

### Patient Characteristics

During the 5-year study period, 617 patients underwent a renal biopsy. Table 1 summarizes their characteristics before and after imputation of missing data. The median age was 57 years (interquartile range: 46-66) and 37% were women. We performed slightly more renal allograft than native biopsies (40% native kidney) and slightly more outpatient than inpatient biopsies (58% outpatient).

### Types and Timing of Bleeding Events

Bleeding events occurred in 79 of 617 patients (12.8%; 95% confidence interval [CI]: 10.4%-15.7%, Table 2). Of these events, 12 were considered major (1.9%; 95% CI: 1.1%-3.4%) with 10 requiring blood transfusion only (1.6%; 95% CI: 0.9%-3.0%) and 2 requiring embolization or surgical intervention (0.3%; 95% CI: 0.1%-1.2%). Major bleeding events occurred twice as frequently in native kidney biopsies than transplant allografts (Table 3).

**Table 2.** Type and Timing of Kidney Biopsy Bleed.

Bleeding characteristics	Number with events	% of all 617 patients	% of 79 patients with a bleed
**Type of bleed**			
**Any**	79	12.8	100.0
**Minor (no intervention)**	67	10.9	84.8
**Major (transfusion only)**	10	1.6	12.7
**Major (surgery or embolization)**	2	0.3	2.5
**Time to event**			
**Immediate**	73	11.8	92.4
**2 hours**	1 (major, transfusion)	0.2	1.3
**4 hours**	1 (minor, no intervention)	0.2	1.3
**5 hours**	1 (major, transfusion)	0.2	1.3
**12 hours**	1 (minor, no intervention)	0.2	1.3
**20 hours**	1 (minor, no intervention)	0.2	1.3
**120 hours**	1 (minor, no intervention)	0.2	1.3

**Table 3.** Bleeding Events in Native vs. Transplant Kidney Biopsies.

**Biopsy target**	**Number of patients**	**Bleeding events**			
		None	Minor	Major (requiring transfusion only)	Major (requiring surgery or embolization)
**Native**	247 (40.0%)	214 (86.6%)	26 (10.5%)	5 (2.0%)	2 (0.8%)
**Transplant**	370 (60.0%)	324 (87.6%)	41 (11.1%)	5 (1.4%)	0

Immediate post-biopsy ultrasound detected 73 of 79 bleeds (92.4%; 95% CI: 84.4%-96.5%). Four minor bleeds were not detected immediately; only 2 patients suffered a major complication that was not recognized immediately after biopsy. These 2 complications occurred among inpatients and required transfusion but no further intervention. These were identified within 2 and 5 hours. There were no nephrectomies or deaths associated with renal biopsies.

### Procedure Characteristics

Most biopsies (90.1%) included 2 (66.0%) or 3 (24.1%) needle passes; no biopsy required more than 5. Table 4 provides a summary of bleeding events by number of needle passes.

**Table 4.** Number of Biopsy Needle Passes and Bleeding Events.

**Number of needle passes**	**Number of patients**	**Bleeding events**			
		None	Minor	Major (requiring transfusion only)	Major (requiring surgery or embolization)
**1**	10	6 (60.0%)	3 (30.0%)	1 (10.0%)	0 (0%)
**2**	407	356 (87.5%)	44 (10.8%)	6 (1.5%)	1 (0.25%)
**3**	149	133 (89.3%)	14 (9.4%)	2 (1.3%)	0 (0%)
**4**	35	31 (88.6%)	4 (11.4%)	0 (0%)	0 (0%)
**5**	16	12 (75.0%)	2 (12.5%)	1 (6.25%)	1 (6.25%)

### Kidney Biopsy Risk Calculator

**Figure 1.**
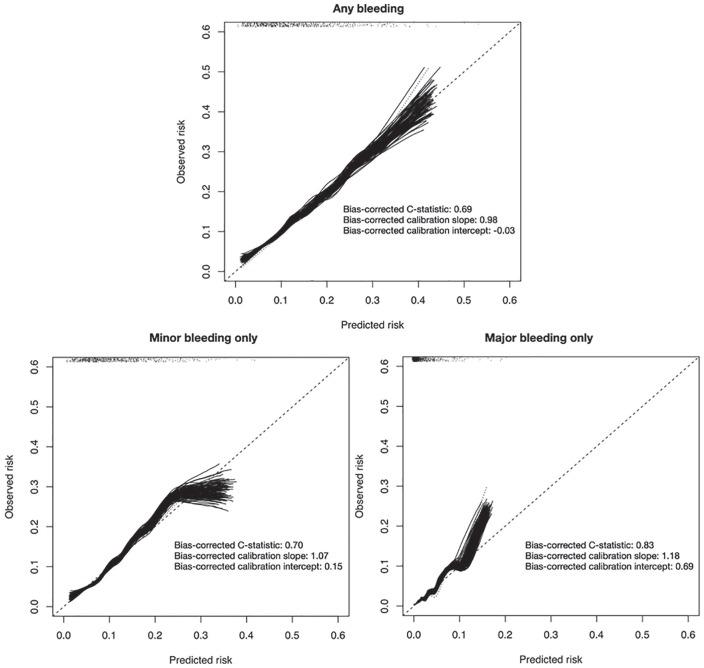
Results of internal validation. *Note.* Results of internal validation in 1,000 bootstrap samples drawn from each of 100 imputed datasets. Calibration curves closer to the diagonal indicate closer agreement between predicted and observed risk. Regions above the diagonal indicate that predictions that are too low; regions below the diagonal indicate predictions that are too high. The rug plot at the top of each graph indicates the distribution of patients across predicted risks derived from the first imputed dataset.

Figure 1 summarizes the results of internal validation for the models for predicting bleeding complications. The model for predicting any bleed (79 events) had modest discrimination (C-statistic, 0.69), and its predictions were closely calibrated to the observed risks. The model for minor events (67 events) performed similarly to the primary model (C-statistic, 0.70), and its predictions were well calibrated to observed risks. The model for major complications (12 events) had very good discrimination (C-statistic, 0.83) and was well calibrated to observed risks except for predicted risks <15% which tended to underestimate observed risk.

**Figure 2.**
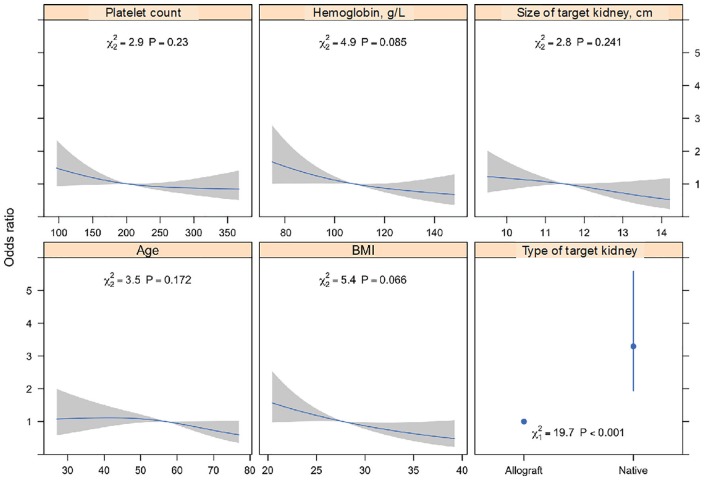
Adjusted associations between predictor variables and development of any bleeding after kidney biopsy. *Note.* Continuous variables were fit using restricted cubic spline functions with 3 knots to allow for nonlinear relationships with the primary outcome. Shaded regions represent 95% CIs. Associations are from 100 imputed datasets and are adjusted for each other in a multivariable model using logistic regression with penalized maximum likelihood estimation.

Figure 2 summarizes the adjusted odds ratios (ORs) relating the predictor variables to the risk of any bleed from the final model. We found bleeding more commonly after biopsies of native kidneys than of allografts (19.4% vs. 8.4%; adjusted OR: 3.3; 95% CI: 1.9%-5.6%). Age, platelet count, pre-procedure hemoglobin, lower BMI, and smaller kidney size on ultrasound all contributed to the risk of bleeding; although none of these factors was statistically significant on its own, their combination improved predictions.

Supplementary Appendix 1 provides the full prediction equations to estimate risk of the composite of any bleeding event and separately for minor and major bleeds. We used the equations to create a risk calculator at http://perioperativerisk.com/kbrc for use on desktop and handheld devices.

## Discussion

We performed a retrospective cohort study of 617 patients who had a percutaneous ultrasound-guided biopsy of a native or transplant kidney performed by Nephrologists and Nephrology trainees at a single academic center in London, Ontario, Canada. Post-biopsy bleeding was common, occurring in 12.8% of patients, but the vast majority of these events were of no clinical significance. Only 1.6% required a blood transfusion and only 0.3% required invasive management. Detection of these events was immediate in the vast majority (92.4%). The risk of complications could be predicted by a combination of patient age, BMI, platelet count, hemoglobin concentration, size of the target kidney, and whether it was a native kidney or an allograft.

A meta-analysis of 9,546 native kidney biopsies in 34 studies found similar risks of complication: 0.9% of patients required transfusion, 0.6% required embolization, 0.3% suffered urinary tract obstruction secondary to hemorrhage, 0.01% underwent nephrectomy, and 0.02% died.^32^ A recent study demonstrated higher overall complication rates than our study (18.7%) but a similar rate of major complications (1.2%), defined as need for blood transfusion or hematoma evacuation.^33^ These investigators routinely conducted repeat ultrasound at 24 hours post procedure, which likely accounts for the higher rate of bleeding through detection of more silent perinephric hematomas. Another recent study including both native and transplant biopsies showed similar frequency of major bleeding events.^24^ This study routinely repeated blood counts and ultrasounds the day after biopsy. This practice of post-biopsy screening is common,^10,20,34^ but our data support the safety of short observation without repeat ultrasound the following day. The 2 patients in our study who had complications not detected at the time of biopsy were both inpatients whose complication was defined by a post-biopsy blood transfusion; with the potential for a lower threshold for transfusion among inpatients and other indications for transfusion, it is possible that these transfusion events were unrelated to the biopsy.

Several studies performed exclusively in patients with native kidney biopsies have demonstrated higher rates of complications than those that include both native and transplant biopsies,^18,20,22,24,35-37^ and others have identified a trend toward higher risk of complications in native kidney biopsies.^9,17,29,38^ This may be because allografts are easier to access during biopsy and if bleeding occurs, the site is easier to compress. This baseline difference in risk for native versus transplant kidney biopsy is accounted for in our risk calculator. Complications may be more frequent in patients with lower BMI because they have less tissue to tamponade bleeding.^11,26^ Small kidney size has long been associated with poor biopsy yield due to widespread sclerosis or fibrosis and increased risk of bleeding^1^; therefore, biopsies are generally avoided in small sclerotic kidneys. We focused on renal length rather than volume or parenchymal thickness because it is a more readily obtained measurement.

### Strengths

Our study included both transplant and native kidney biopsies performed by the Nephrology division at a tertiary care center using standard of care protocols. The inclusion of both transplant and native kidneys was to allow for application of our outcomes to our practice, which includes both. We collected data using *a priori* defined predictors and outcomes, and few patients had missing data. The statistical methods were designed to avoid overfitting and followed methodological recommendations for multivariable prognostic modeling in small datasets,^30^ including selection of predictors based on prior evidence and rationale instead of statistical significance in our dataset, multiple imputation of missing data, flexible modeling of continuous predictors, penalized maximum likelihood estimation for shrinkage of parameter estimates, and internal validation with bootstrapping. Our risk calculator uses commonly known, reliable information and we have made it available at http://perioperativerisk.com/kbrc.

### Limitations

Availability and accuracy of documentation limited our study. Routine practice at our center did not involve repeat imaging or blood work post procedure unless prompted by patient symptoms or hemodynamic instability. This may contribute to ascertainment bias and potentially the exclusion of minor events.

The small number of events precluded us from reliably examining the predictive ability of more variables that may increase risk of bleeding. For example, too few patients had abnormal INRs to analyze precisely. We judged that the additional degrees of freedom required to model the relationship between surrogate markers of renal function (estimated glomerular filtration, serum creatinine, or urea) and bleeding while accounting for interaction with dialysis would risk statistical overfitting more than the expected true gain in prediction performance. Our model performs well without these variables. Pre-procedure systolic blood pressure is associated with bleeding in other studies,^24,36,37,39^ but we were unable to reliably ascertain blood pressure on the day of procedure from the available records.

Routine practice at our center is to withhold antiplatelet agents for 5 to 7 days prior to biopsy. We did not collect data regarding antiplatelet use because we did not intend for our analyses to inform this practice as it is already standard of care. In addition, while we try to withhold antiplatelet agents for 5 to 7 days, if the importance of the biopsy outweighs the risks, the biopsy will be performed while continuing antiplatelet agents given the low risk identified in previous studies.

We also did not collect data on infectious complications or lacerations of other organs; these are less common than bleeding and a much larger cohort would have been necessary to study them reliably.

Although we took steps during the analysis to ensure that our risk calculator will predict bleeding complications outside of our setting, external validation should be performed if the index is to be used widely. Center-specific factors may affect procedure risk; for example, complications may occur more frequently at centers that perform fewer biopsies.^40^ Predictions for major complications are based on only 12 events and may prove inaccurate in independent validation despite our use of shrinkage methods and rigorous internal validation.

## Conclusions

In this retrospective study of 617 patients who had percutaneous ultrasound-guided renal biopsies at one academic center in Canada minor bleeding was common, but significant bleeding requiring intervention was very rare. Only 2 major complications were not recognized immediately after biopsy. These data support the safety of short post-biopsy monitoring for most patients, and based on these results our program has changed its monitoring for patients undergoing outpatient biopsy to 2 hours of supine observation in a monitored setting, and discharge following a repeat ultrasound to rule out active bleeding. A risk calculator can further personalize estimates of complication risk (http://perioperativerisk.com/kbrc).

## Supplemental Material

[Manuscript_R]Reliability_prefrontal_EEG_markers – Supplemental material for Frequency, Timing, and Prediction of Major Bleeding Complications From Percutaneous Renal Biopsy Click here for additional data file. Supplemental material, [Manuscript_R]Reliability_prefrontal_EEG_markers for Frequency, Timing, and Prediction of Major Bleeding Complications From Percutaneous Renal Biopsy by Melissa Schorr, Pavel S. Roshanov, Matthew A. Weir and Andrew A. House in Canadian Journal of Kidney Health and Disease
