# Supplementary material for: Frequency, Timing, and Prediction of Major Bleeding Complications From Percutaneous Renal Biopsy
Source: Can J Kidney Health Dis. 2020 May 25;7:2054358120923527. doi: 10.1177/2054358120923527 (PMC7251654; doi:10.1177/2054358120923527)
Supplement: [Manuscript_R]Reliability_prefrontal_EEG_markers – Supplemental material for Frequency, Timing, and Prediction of Major Bleeding Complications From Percutaneous Renal Biopsy [file [Manuscript_R]Reliability_prefrontal_EEG_markers.pdf]

Supplementary Appendix

to

Schorr M, Roshanov PS, Weir MA, House AA. Frequency, timing, and prediction of bleeding complications from percutaneous renal biopsy. 2018.

Table of Contents

Estimating composite risk of any complication (primary analysis) 2

Estimating risks of minor and major complications separately 3

eFigure 1. Summary of development and internal validation of the Kidney Biopsy Risk Calculator. 4

Equations to predict complications after kidney biopsy 5

Appendix References 6

Estimating composite risk of any complication (primary analysis)

eFigure 1 summarizes our methods of developing and internally validating the model to estimate patients' risk of any biopsy complication. After multiple imputation[1], we fit a model to predict a biopsy complication using logistic regression across 100 imputed datasets. The model included 6 predictors: 5 continuous variables transformed using restricted cubic spline functions with 3 knots (age, body mass index, platelet count, size of the kidney to be biopsied and pre-procedure hemoglobin), and 1 binary variable indicating the whether the kidney was native or an allograft. To limit the risk of statistical overfitting, we entered all predictors into the model simultaneously without performing any selection on the basis of their estimated association with biopsy complications in this dataset. We then performed internal validation in 1000 bootstrap samples drawn with replacement from each of the 100 imputed datasets, refit the model in each bootstrap sample, tested it in the source imputed dataset, and calculated average bias-corrected C-statistics (a measure of a model's ability to discriminate between patients who develop a complication and those who do not), calibration slope, calibration intercept, and calibration curves (measures of how closely predicted risks match the observed probability of a complication) across the 100 imputed datasets.

To correct for any potential overfitting, we then fit and internally validated the final model with the same 6 predictors using logistic regression with penalized maximum likelihood estimation[2]. To do this, we determined the optimal penalty terms that maximize the modified Akaike's Information Criterion in each of the 100 imputed datasets by systematically searching for the optimal penalty terms over a grid of possible penalties from 0 to 10 by increments of 0.1 for penalties for linear terms and 0 to 30 by increments of 0.5 for penalties of non-linear terms. We then calculated the average linear and non-linear penalty terms. We developed the final prediction model by averaging the 100 models developed across the imputed datasets using Rubin's rules for combining the results from multiple imputation and penalizing the model coefficients by the average optimal linear and non-linear penalty terms[1]. We then drew 1000 bootstrap samples with replacement from each of the 100 imputed datasets, refit the model in each bootstrap sample, and calculated the average bias-corrected c-statistic, calibration slope, calibration intercept, and calibration curves for the penalized model.

We compared the validation results for the candidate model and the final (penalized) model to assess the stability of the modelling procedure and the effect of penalization. The validation results for the final model indicate how well we would expect the model to perform in similar patients outside of our study.

Estimating risks of minor and major complications separately

We used the same approach to fit logistic regression models with penalized maximum likelihoods for minor and major complications separately. The only predictor variable for minor complications was the log-odds of any complication (as estimated from the final model in the previous procedure) and the predictor variables for major complications were the pre-biopsy hemoglobin (as a linear function) and the log-odds of any complication. We used this approach to minimize the number of parameters estimated because there are fewer complications of each type.

Equation 1

$$Predicted\ probability\ of\ minor\ complication$$

$$= \frac{1}{1 + exp(-(ß_1 * \log odds_{any\ complication} + intercept))}$$

Equation 2

$$Predicted\ probability\ of\ major\ complication$$

$$= \frac{1}{1 + exp(-(ß_1 * \log odds_{any\ complication} + ß_2 * \text{Hemoglobin} + intercept))}$$

We performed all analyses in R version 3.5.1; Supplementary Appendix 2 contains the analysis code.

eFigure 1. Summary of development and internal validation of the Kidney Biopsy Risk Calculator.

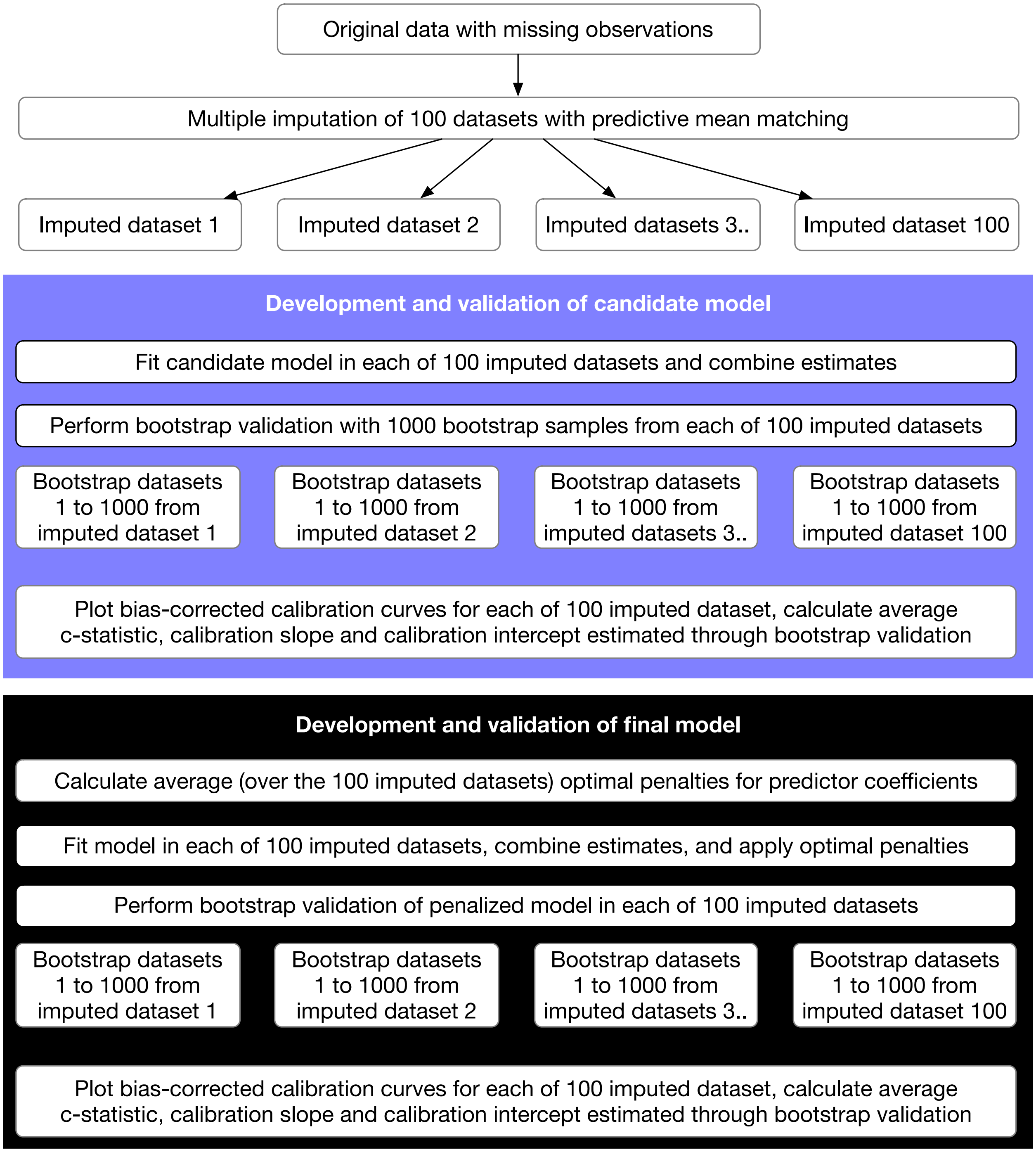

Equations to predict complications after kidney biopsy

Probability of any complication= 1/(1+exp(-(2.7716352+0.0031042236* Age-9.7081209e-06*pmax(Age-31,0)^3+2.4555835e-05*pmax(Age-57,0)^3-1.4847714e-05*pmax(Age-74,0)^3-0.082847525* Size-0.011240109*pmax(Size-10,0)^3+0.019268758*pmax(Size-11.5,0)^3-0.008028649*pmax(Size-13.6,0)^3-0.016588634* PreHg+1.5867369e-06*pmax(PreHg-80,0)^3-3.0199187e-06*pmax(PreHg-108,0)^3+1.4331818e-06*pmax(PreHg-139,0)^3-0.0041121951* Plts+6.334226e-08*pmax(Plts-113.2,0)^3-1.1114171e-07*pmax(Plts-203,0)^3+4.7799453e-08*pmax(Plts-322,0)^3+1.191925*(1 if native)-0.061476145* BMI-8.044627e-06*pmax(BMI-21.39,0)^3+1.3664036e-05*pmax(BMI-27.85,0)^3-5.6194086e-06*pmax(BMI-37.098,0)^3)))

Probability of minor complication = 1/(1+exp(-(-0.32270926+0.92742608*(2.7716352+0.0031042236* Age-9.7081209e-06*pmax(Age-31,0)^3+2.4555835e-05*pmax(Age-57,0)^3-1.4847714e-05*pmax(Age-74,0)^3-0.082847525* Size-0.011240109*pmax(Size-10,0)^3+0.019268758*pmax(Size-11.5,0)^3-0.008028649*pmax(Size-13.6,0)^3-0.016588634* PreHg+1.5867369e-06*pmax(PreHg-80,0)^3-3.0199187e-06*pmax(PreHg-108,0)^3+1.4331818e-06*pmax(PreHg-139,0)^3-0.0041121951* Plts+6.334226e-08*pmax(Plts-113.2,0)^3-1.1114171e-07*pmax(Plts-203,0)^3+4.7799453e-08*pmax(Plts-322,0)^3+1.191925*(1 if native)-0.061476145* BMI-8.044627e-06*pmax(BMI-21.39,0)^3+1.3664036e-05*pmax(BMI-27.85,0)^3-5.6194086e-06*pmax(BMI-37.098,0)^3)))

Probability of major complication = 1/(1+exp(-(1.9745299-0.0406669*PreHg+1.1211287*(2.7716352+0.0031042236* Age-9.7081209e-06*pmax(Age-31,0)^3+2.4555835e-05*pmax(Age-57,0)^3-1.4847714e-05*pmax(Age-74,0)^3-0.082847525* Size-0.011240109*pmax(Size-10,0)^3+0.019268758*pmax(Size-11.5,0)^3-0.008028649*pmax(Size-13.6,0)^3-0.016588634* PreHg+1.5867369e-06*pmax(PreHg-80,0)^3-3.0199187e-06*pmax(PreHg-108,0)^3+1.4331818e-06*pmax(PreHg-139,0)^3-0.0041121951* Plts+6.334226e-08*pmax(Plts-113.2,0)^3-1.1114171e-07*pmax(Plts-203,0)^3+4.7799453e-08*pmax(Plts-322,0)^3+1.191925*(1 if native)-0.061476145* BMI-8.044627e-06*pmax(BMI-21.39,0)^3+1.3664036e-05*pmax(BMI-27.85,0)^3-5.6194086e-06*pmax(BMI-37.098,0)^3)))

Appendix References

1. Marshall A, Altman DG, Holder RL, Royston P: Combining estimates of interest in prognostic modelling studies after multiple imputation: current practice and guidelines. *BMC Med. Res. Methodol.* 9: 57, 2009
2. Moons KGM, Donders a RT, Steyerberg EW, Harrell FE: Penalized maximum likelihood estimation to directly adjust diagnostic and prognostic prediction models for overoptimism: a clinical example. *J. Clin. Epidemiol.* 57: 1262–70, 2004
